# Effects of Chlorhexidine and Sodium Hypochlorite on the Setting Time of Calcium-Enriched Mixture Cement 

**DOI:** 10.7508/iej.2015.03.003

**Published:** 2015-07-01

**Authors:** Mohammad Frough Reyhani, Negin Ghasemi, Sahar Shakouie, Saeed Rahimi, Amin Salem Milani, Babak Ranjbar

**Affiliations:** a *Department of Endodontics, Dental School, Tabriz University of Medical Sciences, Tabriz, Iran; *; b * Dental and Periodontal Research Center, Dental School, Tabriz University of Medical Sciences, Tabriz, Iran; *; c * Private Practice, Tabriz, Iran*

**Keywords:** Calcium-Enriched Mixture, CEM Cement, Chlorhexidine, Setting Time, Sodium Hypochlorite

## Abstract

**Introduction:**

The aim of the present study was to evaluate whether adding 2% chlorhexidine (CHX) and 2.6% sodium hypochlorite (NaOCl) to calcium-enriched mixture (CEM) cement would affect its setting time (ST), or not.

**Methods and Materials:**

In this study, the setting time of CEM cement was evaluated in three groups (*n*=9) as follows: group 1; CEM cement, group 2; CEM cement+2% CHX and group 3; CEM cement+2.6% NaOCl. Then the mean values of ST were calculated and the Kolmogorov-Smirnov test was used to evaluate the normal distribution of data. The Kruskal-Wallis and Mann-Whitney U tests were used for statistical analysis. Statistical significance was set at 0.05.

**Results:**

The mean ST for groups 1, 2 and 3 were 105, 120 and 220 min, respectively. There was a significant increase in the duration of ST in group 3 (NaOCl) in comparison with the two other groups (*P*<0.05).

**Conclusion:**

NaOCl significantly increased the ST of CEM cement, whereas chlorhexidine did not alter the ST.

## Introduction

Different materials have been used to seal the communication pathways between the root canal spaces, the oral cavity and periradicular tissues. Such materials should be biocompatible, antibacterial, non-toxic and radio opaque [1]. Mineral trioxide aggregate (MTA) offers good sealing ability, biocompatibility and non-toxicity, which are favorable in sealing off these communication portals. However, despite its good properties, MTA has a long setting time (ST), difficult handling properties and high cost [2, 3].

Calcium-enriched mixture (CEM) cement is a combination of different compositions of calcium, including calcium oxide, calcium phosphate, calcium carbonate, calcium silicate, calcium sulfate, calcium hydroxide and calcium chloride [1, 2, 4]. The clinical applications of CEM cement are similar to those of MTA and it sets in a moist environment. Some of its properties are superior to MTA such as flow, film thickness and sealing ability. The ST of CEM cement (approximately 50 min) is less than MTA [1, 5], however it is still not suitable for some single-visit treatments and there is need for a second treatment session. On the other hand, when used as a root-end filling material, it might be washed away from the preparation site. 

Attempts have been made to improve the manipulation properties of MTA including its ST. Kogan *et al.* [6] incorporated some chemical compounds into MTA, such as 5% calcium chloride, 2% calcium chloride, 2% lidocaine with 1:100000 epinephrine, KY Jelly, sodium hypochlorite (NaOCl) gel and chlorhexidine (CHX) gel. Among these, 5% calcium chloride, KY Jelly and NaOCl gel decreased the ST of CEM cement. However, 3% calcium chloride had no effect on it. Saline solution and lidocaine did not increase the ST and MTA did not set in the presence of CHX. In addition, it has been shown that incorporation of disodium hydrogen phosphate to MTA decreases its ST [7, 8].

The antibacterial properties of biomaterials such as MTA and CEM cement, have clinical applications in vital pulp therapy and pulp capping. They are also used as retrofilling materials in apicoectomy procedures because lack of profound sealing and bacterial contamination during these procedures can result in treatment failure [9, 10]. Therefore incorporation of bactericidal materials such as CHX and NaOCl into the biomaterials and evaluation of their effects on their physical properties might have clinical relevance. 

The ST is one of the most important properties of a biomaterial; any change in the mixing procedures of hydraulic materials such as CEM cement and MTA might affect the ST [11-13]. Of all the physical properties of CEM cement, only the effect of adding CHX on its push-out bond strength has been evaluated, that decreased its bond strength [14]. Since CEM cement is marketed as a powder and a liquid similar to MTA, liquid compounds such as NaOCl and CHX can be added to its liquid to enable evaluation of their effect on its ST. 

The aim of the present study was to evaluate the effect of incorporating CHX and NaOCl into CEM cement on its ST.

## Materials and Methods

***Preparation of Samples***

According to a previous study by Porter *et al*. [15], a total of 27 samples were prepared for this study in three groups (*n*=9), as follows: group 1; mixture of 1 g of CEM cement powder and 0.36 mL of its liquid according to the manufacturer (Yektazist Dandan, Tehran, Iran), group 2; mixture of 1 g of CEM cement powder plus 0.18 mL of its liquid and 0.18 mL of 2.6% NaOCl and group 3; mixture of 1 g of CEM cement powder plus 0.18 mL of its liquid and 0.18 mL of 2% CHX (Consepsis V, Ultradent Products, Inc., South Jordan, UT, USA).

Before mixing the molds, spatulas and glass slabs were placed at room temperature for 24 h. The mixtures were placed in circular molds measuring 5×15 mm. One g of CEM cement was mixed with any of the three liquids and after mixing for 2 min mixtures were placed in the molds. The samples and the cylinders were placed in an incubator at 37^°^C and 95-100% humidity. After 30 to 60 sec, the setting time was determined with the use of a device for determining the normal consistency and ST of Portland cement (Vicat apparatus, Humboldt Mfg Co., Schiller Park, IL, USA). The special needle of the Vicat machine measured 2±0.1 mm and was inserted into the material surface in a perpendicular manner at a loading rate of 1 mm/min and was kept for 5 sec. The procedure was repeated every 30 sec until the needle was unable to create completely round indentations on the specimen surface. The ST was defined as the time elapsed from the initiation of mixing to the time when the needle was unable to indent the specimen surface, completely.

Data were analyzed using descriptive statistics. The Kolmogorov-Smirnov test was used to evaluate the normal distribution of data. The Kruskal-Wallis test was used to evaluate the significant effect of mixing material on ST; then the Mann-Whitney U test was used for two-by-two comparison of the groups with SPSS software (SPSS version 17.0, SPSS, Chicago, IL, USA). The level of significance was set at 0.05.

## Results

Table 1 presents the mean±SD of ST in study groups. Statistical analysis revealed significant differences in this regard (*P*=0.03). Two-by-two comparison showed a significant increase in the ST in NaOCl group (*P*=0.001), with no significant differences between the two other groups (*P*=0.197).

## Discussion

The present study was designed to evaluate the effect of adding CHX and NaOCl to CEM cement on its ST. The results showed that CHX and NaOCl increased the ST of CEM cement and the effect of NaOCl was more than that of CHX, which was statistically significant. 

The main ingredients of CEM cement are calcium oxide, tricalcium sulfur, calcium phosphate, calcium carbonate, calcium silicate, calcium sulfate, calcium hydroxide and calcium chloride. The clinical applications of CEM cement are similar to those of MTA [5]. 

ST is defined as the time the material requires for setting or hardening from a soft consistency after being mixed [11, 12]. Setting is the minimum strength with which the cement can tolerate its weight. One of the drawbacks of MTA is its long ST, which is approximately 4 h [1]. Long ST increases the number of treatment sessions, making it time-consuming and high cost [6]. A ST of 25-30 min is considered clinically favorable [16]. In fact when a material sets fast, there is limited working time available for its contamination in the oral cavity; on the other hand, the initial strength of the material increases which decreases the odds of material wash-out [17]. Therefore, the restorative material can be placed safely on the set material during the same session [17]. 

Any change in the setting process of bioactive materials, including time and reaction products, predominantly calcium and hydroxyl ions, might affect the formation of hydroxyapatite layer and the bioactivity of these materials [18]. Dimensional changes are reversely affected by the ST; any increase in the ST results in a decrease in dimensional stability [19]. In addition, in some clinical situations a material with a short ST might be necessary. For example, when MTA is used as a root-end filling material, it might be washed away before it sets [8]. Various factors influence the ST of hydraulic biomaterials. 

The amount of liquid used for mixing, the mixing process, the force used for its compaction and the moisture of the environment are factors that affect the ST [20, 21]. In this context, the powder/liquid ratio was similar in all the samples; the materials were placed and condensed by the same operator, and all the samples were stored in the same environment and under the same relative humidity. Another factor is the size of the particles taking part in the setting reaction [22]. Changing the particle sizes might eliminate the need for incorporating different materials to decrease the ST; in many cases adding other materials to decrease the ST compromises the mechanical properties or the biocompatibility of the material. Although particles of CEM cement are smaller than those of MTA, it is inevitable to use CEM cement in two sessions because its ST is almost 1 h [5].

**Table 1:** Mean (SD), and maximum (Max) and minimum (Min) values of setting time (ST) in minutes *(n*=9)

	**Mean (SD) **	**Min ST**	**Max ST**
**CEM cement **	105 (10.60)	90	120.5
**CEM ** **cement+CHX **	120.5 (12.99)	105.5	135
**CEM cement+NaOCl **	220 (25.98)	180	270

The results of studies on the effect(s) of adding NaOCl to MTA were contrary to the results of the present study, showing that NaOCl increased the ST of CEM cement. One of the possible reasons might be the differences between their chemical compositions [5]. Based on a study by Asgary *et al. *[5], calcium oxide is the most abundant constituent in both materials, with a higher percentage in CEM cement. On the other hand, silica is the second most abundant constituent in MTA but it comprises a small percentage of chemical composition of CEM cement. The effect of CHX in increasing the setting time of MTA is similar to that of CEM cement. However, the mechanism of reaction of CHX with MTA is unknown [23]. 

The present results indicate that when CEM cement is used in root-end surgeries and its being washed away is likely due to the long ST, it is advisable not to mix CEM cement with these materials, especially NaOCl. Although the results of the present study in relation to the ST of CEM cement were different from the ST values reported by the manufacturer, in both cases (50 min ST as announced by the manufacturer *vs*. approximately 105 min in the present study) the treatment should be rendered in two sessions and incorporation of CHX and NaOCl will not improve the situation. 

Scanning electron microscopy (SEM) is suggested to evaluate the crystals after setting subsequent to incorporation of these two materials into the liquid of CEM cement.

## Conclusion

Setting time of CEM cement increases after incorporation of NaOCl into its liquid content; however CHX does not alter the setting time.
